# Multi-feature SEIR model for epidemic analysis and vaccine prioritization

**DOI:** 10.1371/journal.pone.0298932

**Published:** 2024-03-01

**Authors:** Yingze Hou, Hoda Bidkhori

**Affiliations:** 1 Department of Industrial Engineering, University of Pittsburgh, Pittsburgh, Pennsylvania, United States of America; 2 Department of Computational and Data Sciences, George Mason University, Fairfax, Virginia, United States of America; Gulf University for Science and Technology, KUWAIT

## Abstract

The SEIR (susceptible-exposed-infected-recovered) model has become a valuable tool for studying infectious disease dynamics and predicting the spread of diseases, particularly concerning the COVID pandemic. However, existing models often oversimplify population characteristics and fail to account for differences in disease sensitivity and social contact rates that can vary significantly among individuals. To address these limitations, we have developed a new multi-feature SEIR model that considers the heterogeneity of health conditions (disease sensitivity) and social activity levels (contact rates) among populations affected by infectious diseases. Our model has been validated using the data of the confirmed COVID cases in Allegheny County (Pennsylvania, USA) and Hamilton County (Ohio, USA). The results demonstrate that our model outperforms traditional SEIR models regarding predictive accuracy. In addition, we have used our multi-feature SEIR model to propose and evaluate different vaccine prioritization strategies tailored to the characteristics of heterogeneous populations. We have formulated optimization problems to determine effective vaccine distribution strategies. We have designed extensive numerical simulations to compare vaccine distribution strategies in different scenarios. Overall, our multi-feature SEIR model enhances the existing models and provides a more accurate picture of disease dynamics. It can help to inform public health interventions during pandemics/epidemics.

## Introduction

This paper develops a new SEIR model to facilitate the epidemic analysis and make appropriate decisions regarding vaccine prioritization and social distancing during pandemics/epidemics. SIR and its variant SEIR have been widely used to analyze the dynamics of epidemics. The classical SIR model classifies people into four states: *susceptible* (vulnerable to disease but not carrying virus), *infected* (symptomatic patient who can spread the virus to others), *recovered* (recovered from the disease), and *dead*. The SEIR (susceptible-exposed-infected-recovered) model is an extension of the SIR model when there is a non-trivial incubation period. It has one additional state, *exposed*, referring to people exposed to the virus but currently asymptomatic.

SIR and SEIR models have been broadly utilized to study the COVID pandemic. We summarize some of the existing works. The spread of COVID among communities using SIR model is investigated in [1]. Besides, short-term predictions based on dynamic regional outbreaks are conducted with SEIR model in [2]. To control the spread of COVID, a study utilizes the SIR model in lockdown policies to control the epidemic [3]. Moreover, the effect of social distancing is evaluated via SEIR model in [4]. Apart from these studies, efforts are also made to extend the SEIR model. SEAIR extracts the *asymptomatic* (A) state from the exposed state to further consider different severeness of symptoms [5]. A discrete-time SEIR model with time-varying parameters is applied for interval prediction, and quarantine influences [6]. Nonetheless, the above models assume homogeneous populations for all aspects, including sensitivity and contact rate.

Besides, the heterogeneous population has been previously studied, and in the following, we review some of the literature. The heterogeneous population is considered by incorporating populations from multiple regions in [2]. However, it assumes a homogeneous population within one region and does not consider cross-regional communication. A SIR model considers heterogeneous social interactive levels of the population with homogeneous sensitivity parameters [7]. Another extended SEIR model applies group-specific sensitivity parameters to classify different severeness of symptoms but uses identical contact rates [8]. Furthermore, reliable estimation of the sensitivity/infection parameters is essential for the effectiveness of SEIR models.

The forecasting quality of SIR models can be affected by the choice of parameter values [9]. The potential prediction bias exists with the use of growth rate in infection from early-days estimation [7]. An evaluation of SIR on COVID finds its poor performance in long-term forecasting due to the parameters not aligning with long-term changes [10]. To enhance the parameter reliability and predictive capabilities, a SEIQR (susceptible-exposed-infected-quarantined-recovered) model incorporates machine learning models to optimize the value of parameters [11]. Besides, some papers study the influential factors in estimation to better predict the spread [12, 13].

Additionally, since the surge of COVID, there have been works on SIR and SEIR models with intervention, including prioritizing vaccines. An adapted SEIR captures the impact of containment measures affecting the infection rate [14]. Nonetheless, it does not discuss the modeling of vaccination. Regarding papers on prioritizing vaccines, they assume the distinction between susceptible and exposed people and vaccinate them separately [9, 15]. In practice, testing is required to distinguish between susceptible and exposed people [16].

Lastly, even though most SEIR models can use time-dependent parameters, they are deterministic within every single period for estimation. The population can change unexpectedly, which makes the pre-determined vaccination plan inefficient against the mass spread of disease. To ensure the efficiency of the vaccination plan against such uncertainty, a chance constraint formulation of the minimization is proposed [17]. Another study ensures that the solution is effective with a high probability [18].

In this paper, we develop a new multi-feature SEIR model with an innovative way to estimate new infections by incorporating heterogeneous population characteristics (contact rates and sensitivities to disease). To ensure estimation reliability, our model uses new infection parameters relating to the form of contact. To demonstrate the prediction quality of our model, we compare the estimated infection cases with the confirmed infections from CDC and present an improvement to the classic SEIR model. Subsequently, we utilize our new model to formulate optimization problems to prioritize vaccines, and we evaluate vaccination strategies for different types of heterogeneous populations. Results show that a strategy similar to the COVID vaccination protocol is not always the most effective under severe situations. Our designed intuition-based vaccination strategy can be as effective as the heuristic solutions to the relevant vaccine optimization problem with the advantage of less computing time. Considering the possibility of uncertainties and underestimation in our vaccine optimization problem, we further develop a chance-constraint optimization problem (CCP) to prioritize vaccines. In some cases, the heuristic solution of CCP can outperform our designed intuition-based vaccination strategies.

This paper is organized as follows. The materials and methods section consists of four subsections. We first review the classic SEIR model. Next, we introduce our new multi-feature SEIR model, where we integrate contact rate and sensitivity to estimate the population changes and allow heterogeneity in these features. Then, we discuss the modeling of vaccine prioritization using our multi-feature SEIR and how to prioritize vaccines within different population states. We formulate vaccine prioritization as an optimization problem. To account for underestimation and uncertainties, we further propose a chance-constraint version of the optimization via Conditional Value-at-Risk (CVaR). Lastly, we provide intuition-based vaccine prioritization strategies as well as optimization-based heuristic algorithms to solve the problem.

The result section consists of four subsections. First, we compare the estimated infections, both from our multi-feature SEIR and classic SEIR model, with the confirmed COVID infections in Allegheny County (Pennsylvania, USA) and Hamilton County (Ohio, USA). Then, we discuss the choice of parameters and performance metrics. Next, we evaluate intuition-based strategies under different severeness of the epidemic. Finally, we compare the performance of our intuition-based strategies with the heuristic solutions of our proposed optimization models. In the discussion section, we summarize and discuss the findings of each numerical study.

## Materials and methods

This section discusses the multi-feature SEIR model with vaccination, and its content is structured as follows. In the first subsection *SEIR model review*, we introduce the classic SEIR model as the foundation of our analysis. In subsection *multi-feature SEIR model*, we present our new model that incorporates contact rates and sensitivity parameters. In subsection *multi-feature SEIR model with vaccine prioritization*, we outline our approach to modeling vaccine prioritization. We formulate corresponding optimization problems to minimize the change in susceptible populations. In the last subsection *intuition-based vaccination and heuristic solutions*, we provide solution approaches to tackle vaccine prioritization effectively.

### SEIR model review

**Fig 1:**
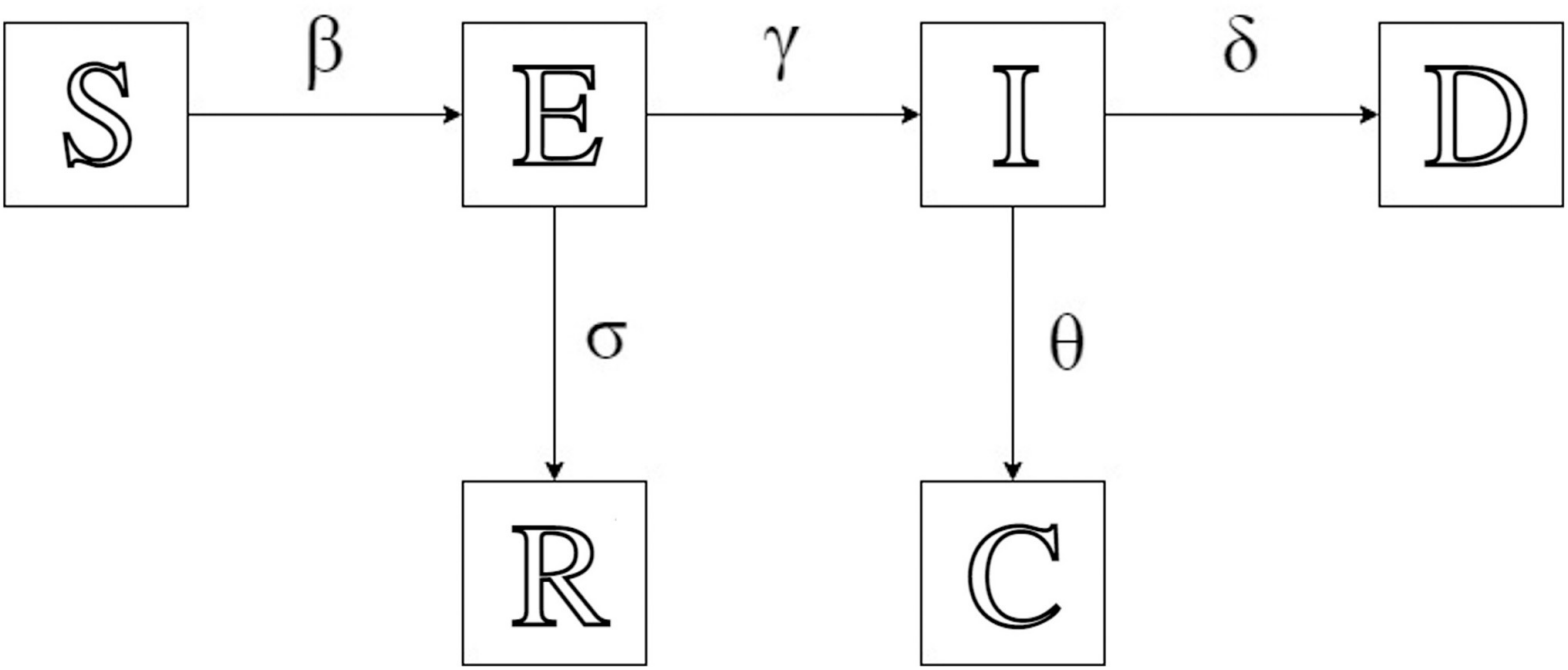
The classic SEIR model. Each state are noted in hollow letters. Their corresponding rate of change to the next state are marked on the arrow.

The SEIR model is an extension of SIR when there is a non-trivial incubation period. It describes the dynamics of infectious diseases by dividing the population into the following different states, Susceptible, Exposed, Infected, Recovered, Cured, and Death [3, 5, 19]: Susceptible (S), uninfected but vulnerable individuals who never encounter or do not carry the virus; Exposed (E), infected but asymptomatic people who carry the virus and can infect others;

Infected (I), patients from state E who developed symptoms; Recovered (R), other people from state E who recovered from the disease before becoming seriously ill; Cured (C), people previously infected but recovered from the disease; Dead (D), infected people who died due to the disease. Note that the difference between recovered and cured is that cured people can be observed since they are previously symptomatic. While recovered people is asymptomatic and therefore not distinguishable from susceptible and exposed people, unless via testing. The total population is the sum of the populations in all states, except for the population in D. Fig 1 shows how population changes in the SEIR model, with different rates of change from one state to another.

The single-direction flow assumes that people can only be infected once. Parameters (rates of change for one predefined period) on the arrows are the sensitivity parameters corresponding to the state a person currently stays in. Effective contact rate/transmission rate (*β*) counts the average number of new infections caused by effective contact (meeting), where virus transmission happens between one infectious individual and one susceptible. Exposed-infected rate (*γ*) is the percentage of exposed people developing symptoms, estimated by the incubation period. Recovery rate for exposed (*σ*) is the percentage of exposed people recovering. It is estimated by the corresponding recovery time. Cured rate for infected (*θ*) is the percentage of infected people recovering. It is also estimated by the corresponding recovery time. Death rate (*δ*) is the percentage of infected people who die due to the disease. It is estimated by case fatality rate, the proportion of deaths compared to the total number of people diagnosed with the disease for a particular period.

The following system of equations summarizes the law of motion for the SEIR model for discrete time. Each term *X*_*t*_ for *X* ∈ {*S*, *E*, *R*, *I*, *C*, *D*} refers to the population of state X∈{S,E,I,R,C,D} at the beginning of the *t*-th period.

$$\begin{array}{c} S_{t+1}=S_{t}-\beta \left(E_{t}+I_{t}\right)S_{t} \end{array}$$ (1a)

$$\begin{array}{c} E_{t+1}=E_{t}+\beta \left(E_{t}+I_{t}\right)S_{t}-\sigma E_{t}-\gamma E_{t} \end{array}$$ (1b)

$$\begin{array}{c} R_{t+1}=R_{t}+\sigma E_{t} \end{array}$$ (1c)

$$\begin{array}{c} I_{t+1}=I_{t}+\gamma E_{t}-\theta I_{t}-\delta I_{t} \end{array}$$ (1d)

$$\begin{array}{c} C_{t+1}=C_{t}+\theta I_{t} \end{array}$$ (1e)

$$\begin{array}{c} D_{t+1}=D_{t}+\delta I_{t} \end{array}$$ (1f)

$$\begin{array}{c} N_{t+1}=N_{t}-\delta I_{t} \end{array}$$ (1g)

We use *N*_*t*_ to denote the total alive population during *t*-th period. We assume that effective contact happens only between S and E people in (1a) in the rest of our discussion if infected individuals can be quarantined. A detailed explanation can also be found in [3, 5].

The classic SEIR model allows both continuous and discrete change for prediction. Nonetheless, it assumes homogeneous individuals, regardless of different demographic features that can affect the epidemic. This also affects the analysis of testing or vaccination on different people. Therefore, we extend the SEIR by considering multiple features of the population to provide a more accurate prediction of infection. Moreover, we propose a new vaccination prioritization model for heterogeneous populations using the framework of multi-feature SEIR for effective control of the disease.

### Multi-feature SEIR model

This subsection extends the classic SEIR model to consider different social activity levels (contact rate) and health conditions (sensitivity). Moreover, we modify the estimation of newly exposed people using new infection parameters, contact rate, and sensitivity, which can be different across the population. Existing models estimate new exposed people using *β* in (1a). This parameter is estimated by a state population and assumes uniform behavior [12]. It is also difficult to distinguish different sensitivities using *β* [20].

**Fig 2:**
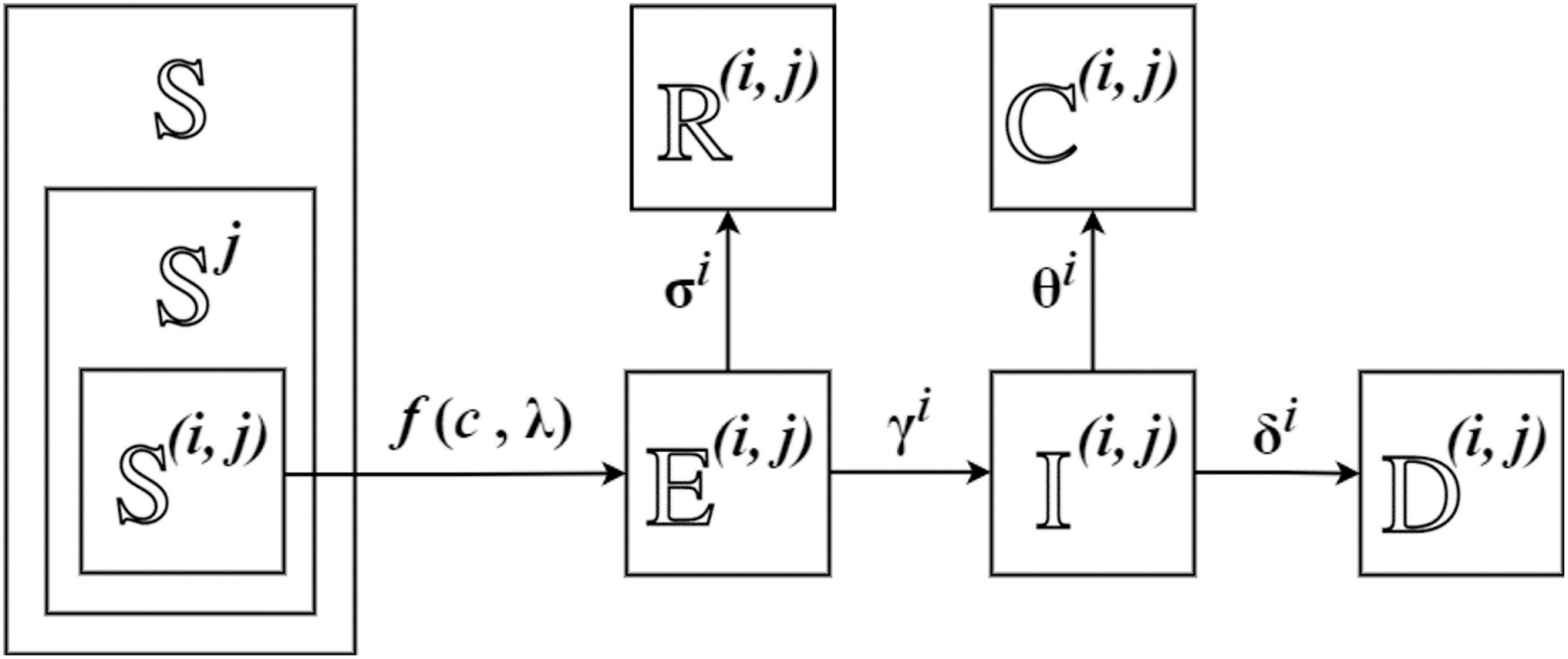
Multi-feature SEIR model. Population is classified into difference (*i*, *j*) divisions, with sensitivity *s*^*i*^ and contact rate *c*^*j*^.

We consider the extension of the classic SEIR model by adapting different contact rates and sensitivity (rates of change) [21]. Fig 2 shows the dynamics of the new multi-feature SEIR model, where we use (*i*, *j*) division to distinguish different people with sensitivity *s*^*i*^ and contact rate *c*^*j*^. Within each division, people are assumed to be identical. The term $X_{t}^{\left(i,j\right)}$ stands for the people of state X in (*i*, *j*) division during *t*-th period. *f*(*c*, λ) plays a similar role to *β*, representing the rate of change for susceptible people. It depends on both contact rate *c* and probability of infection of close contact λ. The *f*(*c*, λ) function is the right-hand side of (2). Our new model considers a new infection parameter λ. The calculation of λ considering the form of contact, cough volume, distance, and other related factors has been discussed in [22]. We assume uniform λ across the population since it only depends on the form of contact and is irrelevant to health condition [22, 23]. We allow λ to be time-varying due to the contact form can be frequently affected by the variants of the virus, people, and regulations [22]. The rest of the notations are explained in Table 1.

**Table 1:** Notations for multi-feature SEIR model.

**Sets and Population**	
$S_{t}$	Susceptible people (population *S*_*t*_) at the beginning of *t*-th period.
$E_{t}$	Exposed people (population *E*_*t*_) at the beginning of *t*-th period.
$R_{t}$	Recovered people (population *R*_*t*_) at the beginning of *t*-th period.
$I_{t}$	Infected people (population *I*_*t*_) at the beginning of *t*-th period.
$C_{t}$	Cured people (population *C*_*t*_) at the beginning of *t*-th period.
$D_{t}$	Cumulative death (population *D*_*t*_) at the beginning of *t*-th period.
X	General notation for population state X∈{S,E,I,R,C,D}.
**Parameters and Indices**	
*t*	Index for period starting at *t*, with total number of *T*.
*β*	Average number of new infection per contact (virus-transmission).
λ	Infection probability from susceptible to exposed
*γ*	Exposed-infected rate
*σ*	Recovery rate for exposed
*θ*	Recovery rate for infected
*δ*	Death rate for infected
*s*, *s*^*i*^, *s*^*m*^	Sensitivity *s* ∈ {λ, *γ*, *σ*, *θ*, *δ*} with *i*, *m* = 1, ⋯, *M*.
*c*, *c*^*j*^, *c*^*k*^	Contact rate with *j*, *k* = 1, ⋯, *K*.
(*i*, *j*), (*m*, *k*)	Population division with *s*^*i*^ (or *s*^*m*^) and *c*^*j*^ (or *c*^*k*^).
$p_{t}^{s}$	Population proportions w.r.t. sensitivity of *t*-th period, $p_{t}^{s}=\left(p_{t}^{s,1},\ldots ,p_{t}^{s,M}\right)$
$p_{t}^{c}$	Population proportions w.r.t. contact rate of *t*-th period, $p_{t}^{c}=\left(p_{t}^{c,1},\ldots ,p_{t}^{c,K}\right)$
**Sub-populations**	
$X_{t}^{i}$ , $X_{t}^{m}$	People in state X with sensitivity *s*^*i*^ (or *s*^*m*^) at the beginning of *t*-th period, with population $X_{t}^{i}$ (or $X_{t}^{m}$).
$X_{t}^{j}$ , $X_{t}^{k}$	People in state X with contact rate *c*^*j*^ (or *c*^*k*^) at the beginning of *t*-th period, with population $X_{t}^{j}$ (or $X_{t}^{k}$).
$X_{t}^{\left(i,j\right)}$	People in state X of (*i*, *j*) at the beginning of *t*-th period, with population $X_{t}^{\left(i,j\right)}$.

Eq (2) is alternative to (1a) in classic SEIR. Instead of using *β*, we use infection probability for close contact, contact rate, and sensitivity to estimate the population change for state S and E. The population change for $S_{t}^{\left(i,j\right)}$ regarding contacts with all exposed people is estimated as follows:

$$\begin{array}{c} S_{t+1}^{\left(i,j\right)}-S_{t}^{\left(i,j\right)}=-\lambda \frac{S_{t}^{\left(i,j\right)}}{N_{t}-I_{t}}\sum_{k=1}^{K}c^{k}E_{t}^{k}\;i=1,\cdots ,M\;j=1,\cdots ,K. \end{array}$$ (2)

To justify (2), we first consider the contact between $S_{t}^{\left(i,j\right)}$ and $E_{t}^{\left(m,k\right)}$. The number of contact happening is:

$$\begin{array}{c} C\left(S_{t}^{\left(i,j\right)},E_{t}^{\left(m,k\right)}\right)=E_{t}^{\left(m,k\right)}\cdot c^{k}\cdot p\left(S_{t}^{\left(i,j\right)}\right)=E_{t}^{\left(m,k\right)}\cdot c^{k}\cdot \frac{S_{t}^{\left(i,j\right)}}{N_{t}-I_{t}}. \end{array}$$ (3)

Note that this is an estimation for contacted susceptible people, which has been used in [24]. $E_{t}^{\left(m,k\right)}$ is the number of exposed population in (*m*, *k*) division with sensitivity *s*^*m*^ and contact rate *c*^*k*^ during *t*-th period. *c*^*k*^ is the average number of different people met by an exposed person in (*m*, *k*) division for a predefined period. Its value can be estimated via social network simulation [25–27]. People in $E_{t}^{\left(m,k\right)}$ make $c^{k}E_{t}^{\left(m,k\right)}$ number of contacts. Among these contacted people, approximately $p(S_{t}^{\left(i,j\right)})$ of them belong to state $S_{t}^{\left(i,j\right)}$. The $p(S_{t}^{\left(i,j\right)})$ denotes the proportion of $S_{t}^{\left(i,j\right)}$ among the current total population (*N*_*t*_ − *I*_*t*_), assuming infected people in quarantine.

Thus, we can calculate the newly exposed people in (*i*, *j*) division for *t*-th period, which is the number of people leaving $S_{t}^{\left(i,j\right)}$. It is estimated by the number of contact happening multiplied by infection probability λ:

$$\begin{array}{c} S_{t+1}^{\left(i,j\right)}-S_{t}^{\left(i,j\right)}=-\sum_{k=1}^{K}\sum_{m=1}^{M}\lambda \cdot C\left(S_{t}^{\left(i,j\right)},E_{t}^{\left(m,k\right)}\right). \end{array}$$ (4)

A negative sign is due to people leaving $S_{t}^{\left(i,j\right)}$. λ is the infection probability measuring the possibility of virus transmission between an exposed and a susceptible person [22]. Eq (4) estimates new exposed in an analogous way to (1a). In (1a), *E*_*t*_*S*_*t*_ estimates all possible contacts (*I*_*t*_ is ignored by quarantine assumption). The term *β* ⋅ *E*_*t*_*S*_*t*_ gives the number of newly exposed people who get virus-transmitted in effective contact. Similarly, Eq (4) estimates the contact number by $C(S_{t}^{\left(i,j\right)},E_{t}^{\left(m,k\right)})$. The λ calculates the average number of effective contact (virus transmitted to a susceptible person) happening per contact among $C(S_{t}^{\left(i,j\right)},E_{t}^{\left(m,k\right)})$. The summation is over sensitivity index *m* and contact index *k*, because the change for $S_{t}^{\left(i,j\right)}$ is caused by contacts with exposed people from every (*m*, *k*) division. $E_{t}^{k}$, the population of exposed people with contact rate *c*^*k*^, is calculated by Eq (5), which is also suitable for other states and total population *N*:

$$\begin{array}{c} X_{t}^{k}=\sum_{m=1}^{M}X_{t}^{\left(m,k\right)},\;k=1,\cdots ,K,\;X\in \left\{S,E,I,R,C,D,N\right\}. \end{array}$$ (5)

The basic reproduction number, *R*_0_(*t*), can be estimated following the rational of Eq (2). *R*_0_(*t*) quantifies the expected number of new cases generated by a single case at a given time *t*, where all individuals are susceptible to infection, with the assumption that no other individuals are infected or immunized [28, 29]. The *R*_0_(*t*) can be estimated by:

$$\begin{array}{c} R_{0}\left(t\right)=\sum_{k=1}^{K}p_{t}^{c,k}(\sum_{i,j}\lambda \frac{S_{t}^{\left(i,j\right)}}{N_{t}-I_{t}}c^{k}). \end{array}$$ (6)

The term $\sum_{i,j}\lambda \frac{S_{t}^{\left(i,j\right)}}{N_{t}-I_{t}}c^{k}$ estimates the new cases resulting from a single exposed individual with a contact rate of *c*^*k*^. We take the average with respect to $p_{t}^{c,k}$, the proportion of people with contact rate *c*^*k*^ at time *t*.

For population change of other states in (*i*, *j*) division, corresponding changes are made to the exposed state, while changes in other states remain the same as the classic SEIR model in (1c) to (1g), except for replacing *X*_*t*_ by $X_{t}^{\left(i,j\right)}$:

$$\begin{array}{c} E_{t+1}^{\left(i,j\right)}=E_{t}^{\left(i,j\right)}+\lambda \sum_{k=1}^{K}c^{k}\frac{S_{t}^{\left(i,j\right)}}{N_{t}-I_{t}}E_{t}^{k}-\sigma_{i}E_{t}^{\left(i,j\right)}-\gamma_{i}E_{t}^{\left(i,j\right)},\\ R_{t+1}^{\left(i,j\right)}=R_{t}^{\left(i,j\right)}+\sigma_{i}E_{t}^{\left(i,j\right)},\\ I_{t+1}^{\left(i,j\right)}=I_{t}^{\left(i,j\right)}+\gamma_{i}E_{t}^{\left(i,j\right)}-\theta_{i}I_{t}^{\left(i,j\right)}-\delta_{i}I_{t}^{\left(i,j\right)},\\ C_{t+1}^{\left(i,j\right)}=C_{t}^{\left(i,j\right)}+\theta_{i}I_{t}^{\left(i,j\right)},\\ D_{t+1}^{\left(i,j\right)}=D_{t}^{\left(i,j\right)}+\delta_{i}I_{t}^{\left(i,j\right)},\\ N_{t+1}^{\left(i,j\right)}=N_{t}^{\left(i,j\right)}-\delta_{i}I_{t}^{\left(i,j\right)}. \end{array}$$

### Multi-feature SEIR model with vaccine prioritization

In this subsection, we utilize the multi-feature SEIR to model vaccine prioritization among different population groups/divisions. We assume that vaccinated people will no longer be infected, and the vaccine will take effect in the next coming period. Our model defines a new variable to measure the proportion of vaccinated people among asymptomatic people (susceptible, exposed, recovered). We give the final system of equations of our multi-feature SEIR with vaccine prioritization. Lastly, we define a chance-constraint optimization problem in response to the difference between reality and estimation.

#### Vaccine prioritization modeling

To model vaccination, we add a new state Immunized (V), to indicate immunized people after vaccination. A decision variable $v_{t}^{\left(i,j\right)}$ is defined to measure the proportion of vaccinated people among asymptomatic people (susceptible, exposed, and recovered) in (*i*, *j*) division. We assume that asymptomatic people in the same division are equally likely to get vaccinated. The vaccination of infected and cured people is not considered in this paper, since their population is observable and much easier for planning. But it can be adjusted to our model by the cured rate for infected people (*θ* or *θ*_*i*_). In the following, we discuss the population change of each state for (*i*, *j*) division.

The following equation explains the susceptible population change regarding contact with exposed people of all contact rates and sensitivity under vaccination:

$$\begin{array}{c} S_{t+1}^{\left(i,j\right)}=S_{t}^{\left(i,j\right)}-v_{t}^{\left(i,j\right)}S_{t}^{\left(i,j\right)}-\Delta S_{t}^{\left(i,j\right)}. \end{array}$$ (7)

$v_{t}^{\left(i,j\right)}$ is the vaccination coverage ratio for people in (*i*, *j*) division during the *t*-th period of time. $\Delta S_{t}^{\left(i,j\right)}$ represents the population of virus-transmitted, unvaccinated people, who will be in state $E_{t+1}$ for the next period.

**Fig 3:**
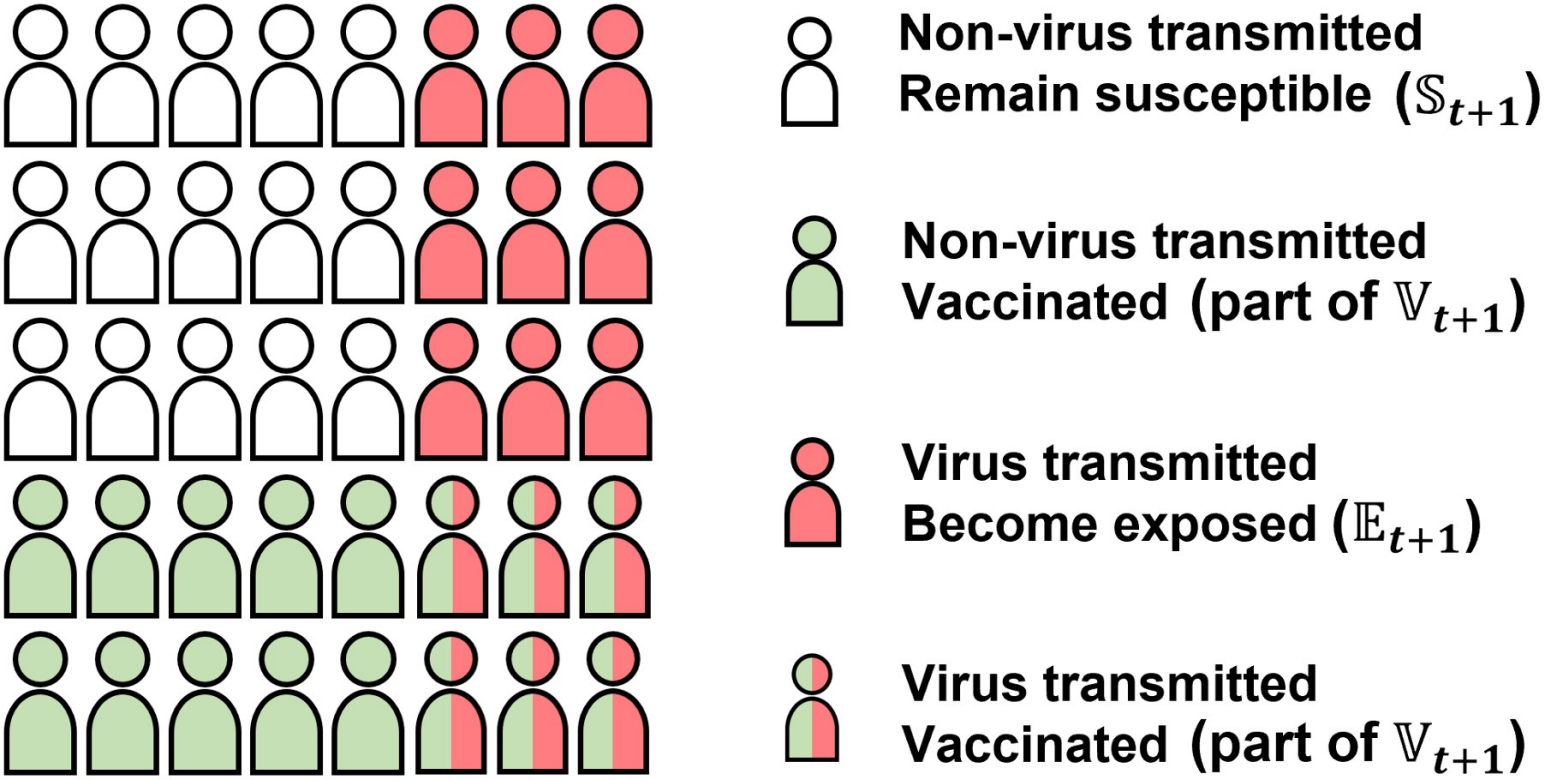
Population changes for *S*_*t*_ population. All four kinds of people are susceptible at time *t*. White people have no changes and remain susceptible for time *t* + 1. Green people get vaccinated during this time and are not exposed to the virus. Red people get virus-transmitted from other virus carriers and do not receive vaccination. Green-red people get vaccinated and get virus-transmitted. But they are treated as vaccinated people with immunity, and will not be counted as exposed for time *t* + 1.

Fig 3 illustrates the distinction of non-virus-transmitted people, virus-transmitted and vaccinated people, virus-transmitted and unvaccinated people.

Consider the *t*-th period, for the people in state S, there are four possible changes to them. First, there are people not receiving the virus and remaining susceptible for *t* + 1. Second, there are people not getting virus transmitted and getting vaccinated, who will be in state $V_{t+1}$ for the next period. Moreover, there are virus-transmitted susceptible people, whose population is estimated by Eq (2). Some of them are vaccinated to be in state $V_{t+1}$. The remaining unvaccinated people, whose population is *ΔS*_*t*_, will be in state $E_{t+1}$. The population of all vaccinated susceptible people is estimated by Eq (8), which is part of $V_{t+1}$:

$$\begin{array}{c} v_{t}^{\left(i,j\right)}\cdot \left(S_{t}^{\left(i,j\right)}+E_{t}^{\left(i,j\right)}+R_{t}^{\left(i,j\right)}\right)\cdot \frac{S_{t}^{\left(i,j\right)}}{S_{t}^{\left(i,j\right)}+E_{t}^{\left(i,j\right)}+R_{t}^{\left(i,j\right)}}=v_{t}^{\left(i,j\right)}S_{t}^{\left(i,j\right)}, \end{array}$$ (8)

where $v_{t}^{\left(i,j\right)}$ indicates the vaccinated proportion of all asymptomatic population $\left(S_{t}^{\left(i,j\right)}+E_{t}^{\left(i,j\right)}+R_{t}^{\left(i,j\right)}\right)$, and $S_{t}^{\left(i,j\right)}/\left(S_{t}^{\left(i,j\right)}+E_{t}^{\left(i,j\right)}+R_{t}^{\left(i,j\right)}\right)$ of them are susceptible on average. Similarly, the population of state E and R who get vaccinated is estimated by $v_{t}^{\left(i,j\right)}E_{t}^{\left(i,j\right)}$ and $v_{t}^{\left(i,j\right)}R_{t}^{\left(i,j\right)}$, respectively.

Using the ratio $v_{t}^{\left(i,j\right)}$, Eq (9) estimates $\Delta S_{t}^{\left(i,j\right)}$, the population of new exposed people for the next period, who are unvaccinated and virus-transmitted S people:

$$\begin{array}{cc} \Delta S_{t}^{\left(i,j\right)}=(1-v_{t}^{\left(i,j\right)})\sum_{m,k}\lambda \;C(S_{t}^{\left(i,j\right)},E_{t}^{\left(m,k\right)})\\ =(1-v_{t}^{\left(i,j\right)})\lambda \sum_{k=1}^{K}\sum_{m=1}^{M}c^{k}\frac{S_{t}^{\left(i,j\right)}}{N_{t}-I_{t}}E_{t}^{\left(m,k\right)}\\ & =(1-v_{t}^{\left(i,j\right)})\lambda \frac{S_{t}^{\left(i,j\right)}}{N_{t}-I_{t}}\sum_{k=1}^{K}c^{k}E_{t}^{k},\;i=1,\cdots ,M\;j=1,\cdots ,K. \end{array}$$ (9)

We assume vaccine becomes effective in the next period. Thus, the population $S_{t}^{\left(i,j\right)}$ and $E_{t}^{\left(m,k\right)}$ remain unchanged, making $C(S_{t}^{\left(i,j\right)},E_{t}^{\left(m,k\right)})$ the same as Eq (3).

As we consider all asymptomatic people, $v_{t}^{\left(i,j\right)}$ is also applied for state E and R. Based on (1b), the change for population of $E_{t}^{\left(i,j\right)}$ under vaccination is:

$$\begin{array}{c} E_{t+1}^{\left(i,j\right)}=E_{t}^{\left(i,j\right)}+\Delta S_{t}^{\left(i,j\right)}-(1-v_{t}^{\left(i,j\right)})(\sigma_{i}+\gamma_{i})E_{t}^{\left(i,j\right)}-v_{t}^{\left(i,j\right)}(E_{t}^{\left(i,j\right)}+\Delta S_{t}^{\left(i,j\right)}). \end{array}$$ (10)

The term $(1-v_{t}^{\left(i,j\right)})(\sigma_{i}+\gamma_{i})E_{t}^{\left(i,j\right)}$ is the number of unvaccinated people leaving state E (becoming I or R). The last term $v_{t}^{\left(i,j\right)}(E_{t}^{\left(i,j\right)}+\Delta S_{t}^{\left(i,j\right)})$ represents vaccinated population (including $\Delta S_{t}^{\left(i,j\right)}$, for their joining state E). Based on (1c), the population change for $R_{t}^{\left(i,j\right)}$ under same vaccination coverage ratio $v_{t}^{\left(i,j\right)}$ is:

$$\begin{array}{c} R_{t+1}^{\left(i,j\right)}=(1-v_{t}^{\left(i,j\right)})[R_{t}^{\left(i,j\right)}+(1-v_{t}^{\left(i,j\right)})\sigma_{i}E_{t}^{\left(i,j\right)}]. \end{array}$$ (11)

The term $\left(1-v_{t}^{\left(i,j\right)}\right)\sigma_{i}^{E}E_{t}^{\left(i,j\right)}$ represents the population becoming recovered from state E in Eq (10). We vaccinate $v_{t}^{\left(i,j\right)}$ proportion of recovered people, leaving the remaining proportion in state R. Lastly, the effect of vaccine is also reflected in the change in the infected population. Based on Eq (1d), we have the following change for the infected population:

$$\begin{array}{c} I_{t+1}^{\left(i,j\right)}=I_{t}^{\left(i,j\right)}+(1-v_{t}^{\left(i,j\right)})\gamma_{i}E_{t}^{\left(i,j\right)}-\theta_{i}I_{t}^{\left(i,j\right)}-\delta_{i}I_{t}^{\left(i,j\right)}. \end{array}$$ (12)

We consider vaccinating people in states S, E, and R. Vaccination and other medical treatment of people in state I can be reflected in the cured rate for infected, *θ*_*i*_, and it is out of the scope of this paper.

With given population of all states during *t*-th period, for different sensitivity *i* = 1, ⋯, *M* and different contact rate *j* = 1, ⋯, *K*, our multi-feature SEIR model with vaccination gives the dynamics of population changes for each (*i*, *j*) division:

$$\begin{array}{c} \Delta S_{t}^{\left(i,j\right)}=\text{min}\{(1-v_{t}^{\left(i,j\right)})\lambda \frac{S_{t}^{\left(i,j\right)}}{N_{t}-I_{t}}\sum_{k=1}^{N}c^{k}E_{t}^{k},(1-v_{t}^{\left(i,j\right)})S_{t}^{\left(i,j\right)}\}, \end{array}$$ (13a)

$$\begin{array}{c} S_{t+1}^{\left(i,j\right)}=(1-v_{t}^{\left(i,j\right)})S_{t}^{\left(i,j\right)}-\Delta S_{t}^{\left(i,j\right)},\; \end{array}$$ (13b)

$$\begin{array}{c} E_{t+1}^{\left(i,j\right)}=(1-v_{t}^{\left(i,j\right)})[E_{t}^{\left(i,j\right)}+\Delta S_{t}^{\left(i,j\right)}-(\sigma_{i}+\gamma_{i})E_{t}^{\left(i,j\right)}], \end{array}$$ (13c)

$$\begin{array}{c} R_{t+1}^{\left(i,j\right)}=(1-v_{t}^{\left(i,j\right)})[R_{t}^{\left(i,j\right)}+(1-v_{t}^{\left(i,j\right)})\sigma_{i}E_{t}^{\left(i,j\right)}], \end{array}$$ (13d)

$$\begin{array}{c} I_{t+1}^{\left(i,j\right)}=I_{t}^{\left(i,j\right)}+(1-v_{t}^{\left(i,j\right)})\gamma_{i}E_{t}^{\left(i,j\right)}-\theta_{i}I_{t}^{\left(i,j\right)}-\delta_{i}I_{t}^{\left(i,j\right)}, \end{array}$$ (13e)

$$\begin{array}{c} C_{t+1}^{\left(i,j\right)}=C_{t}^{\left(i,j\right)}+\theta_{i}I_{t}^{\left(i,j\right)}, \end{array}$$ (13f)

$$\begin{array}{c} D_{t+1}^{\left(i,j\right)}=D_{t}^{\left(i,j\right)}+\delta_{i}I_{t}^{\left(i,j\right)}, \end{array}$$ (13g)

$$\begin{array}{c} N_{t+1}^{\left(i,j\right)}=N_{t}^{\left(i,j\right)}-\delta_{i}I_{t}^{\left(i,j\right)}, \end{array}$$ (13h)

$$\begin{array}{c} V_{t+1}^{\left(i,j\right)}=V_{t}^{\left(i,j\right)}+v_{t}^{\left(i,j\right)}(S_{t}^{\left(i,j\right)}+E_{t}^{\left(i,j\right)}+R_{t}^{\left(i,j\right)}), \end{array}$$ (13i)

$$\begin{array}{c} X_{t}^{j}=\sum_{i=1}^{M}X_{t}^{\left(i,j\right)},\;X_{t}=\sum_{j=1}^{K}X_{t}^{j},\;X\in \left\{S,E,I,R,C,D,N\right\}. \end{array}$$ (13j)

Eq (13a) ensures the non-negativity of susceptible population. Since other sensitivity parameters are far less than 1, other states are guaranteed to be non-negative all the time in (13c)-(13h). Eq (13i) gives the cumulative vaccinated population. Eq (13j) calculates the population with same contact rate and total population for each state.

#### Optimization formulation

With a given number of vaccines for each period, we can formulate the vaccination prioritization problem as an optimization problem using the multi-feature SEIR model with vaccination. To decide the best practical vaccination strategy, we minimize the summation of $\Delta S_{t}^{\left(i,j\right)}$ from (9), the population of new exposed people over all *i*, *j* and all time *t*, subject to constraints on the amount of available vaccine and multi-feature SEIR model. This number is responsible for the latent infection, as well as all exposed, infected, and death. The optimal solution is a sequence of $v_{t}^{\left(i,j\right)}$ for all *t*, *i*, *j*, deciding the vaccine coverage ratio for each population division in each period. For given population of each state and $V_{t}^{min},V_{t}^{max}$ for all *t*, the optimization is as follows:

$$\begin{array}{cccc} 3\underset{{\left\{v_{t}^{\left(i,j\right)}\right\}}_{t=0}^{T-1}}{\text{min}}\; & \sum_{t=0}^{T-1}\sum_{i=1}^{M}\sum_{j=1}^{K}\Delta S_{t}^{\left(i,j\right)} & & \end{array}$$ (14a)

$$\begin{array}{ccc} s.t.\; & X_{t}^{\left(i,j\right)}=p_{t}^{\left(i,j\right)}X_{t}\;X\in \left\{S,E,I,R,C,D,N\right\}, & t=0 \end{array}$$ (14b)

$$\begin{array}{ccc} & \text{multi-feature}\;\text{SEIR}\;\text{constraints}\;\text{(}13\text{a})\;-(13\text{j}) & t=0,\cdots ,T-1, \end{array}$$ (14c)

$$\begin{array}{ccc} & V_{t}^{min}\leq \sum_{j=1}^{K}\sum_{i=1}^{M}v_{t}^{\left(i,j\right)}(S_{t}^{\left(i,j\right)}+E_{t}^{\left(i,j\right)}+R_{t}^{\left(i,j\right)})\leq V_{t}^{max}\; & t=0,\cdots ,T-1, \end{array}$$ (14d)

$$\begin{array}{ccc} & v_{t}^{\left(i,j\right)}\in \left[0,1\right], & t=0,\cdots ,T-1 \end{array}$$ (14e)

$$\begin{array}{ccc} & X_{t}^{\left(i,j\right)}\geq 0 & t=0,\cdots ,T-1. \end{array}$$ (14f)

Constraint (14b) gives the population of each (*i*, *j*) division in each state at the beginning, where $p_{0}^{\left(i,j\right)}$ is the initial proportion of (*i*, *j*) division in total population of all states. Constraint (14d) represents the vaccination requirement for each period, with the minimum vaccination requirement $V_{t}^{min}$ and maximum available vaccine $V_{t}^{max}$. It is estimated by $p_{0}^{\left(i,j\right)}=p_{0}^{s,i}\cdot p_{0}^{c,j}$, with the proportions defined in Table 1. Constraint (14e) and (14f) are the practical constraints on vaccine coverage ratio and population being non-negative.

#### Chance-constraint optimization

The previous discussion assumes $\Delta S_{t}^{\left(i,j\right)}$ following the estimation of Eq (9), resulting in a static model for each period. Since the actual change in population can deviate from our model estimation, a chance-constraint optimization problem is defined using Conditional Value-at-Risk (CVaR). First, we explain its intuition. Then, we propose corresponding constraints to the optimization.

In reality, the value of $\Delta S_{t}^{\left(i,j\right)}$ is stochastic, following some distribution with probability distribution function $f_{D}\left(\Delta S_{t}^{\left(i,j\right)}\right)$. For the remaining discussion, we must distinguish the actual and estimated value of $\Delta S_{t}^{\left(i,j\right)}$. Denote the actual value of $\Delta S_{t}^{\left(i,j\right)}$ by $\Delta {\overset{ˇ}{S}}_{t}^{\left(i,j\right)}∼f_{D}\left(\Delta S_{t}^{\left(i,j\right)}\right)$. The following abbreviation represents our estimation of $\Delta S_{t}^{\left(i,j\right)}$:

$$\begin{array}{c} \Phi_{t}^{\left(i,j\right)}\text{min}\{(1-v_{t}^{\left(i,j\right)})\lambda \frac{S_{t}^{\left(i,j\right)}}{N_{t}-I_{t}}\sum_{k=1}^{N}c^{k}E_{t}^{k},(1-v_{t}^{\left(i,j\right)})S_{t}^{\left(i,j\right)}\}. \end{array}$$

If fewer people are affected, namely $\Delta {\overset{ˇ}{S}}_{t}^{\left(i,j\right)}\leq \Phi_{t}^{\left(i,j\right)}$, the vaccination plan is still efficient since there is sufficient vaccine. However, if $\Delta {\overset{ˇ}{S}}_{t}^{\left(i,j\right)}>\Phi_{t}^{\left(i,j\right)}$, we underestimate the situation, as well as the amount of vaccine needed, making the solution provided by Optimization (14) inefficient.

To ensure the effectiveness of $\Phi_{t}^{\left(i,j\right)}$ and our proposed solution under most situations, one approach is adding a probabilistic constraint [18]:

$$\begin{array}{c} P(\Phi_{t}^{\left(i,j\right)}\geq \Delta {\overset{ˇ}{S}}_{t}^{\left(i,j\right)})\geq \alpha . \end{array}$$ (15)

Namely, with the probability at least *α*, we want our estimation $\Phi_{t}^{\left(i,j\right)}$ to be conservative (more than the actual amount), making our vaccination plan sufficient. The mathematical formulation of (15) is done via Value-at-Risk (VaR) [30]:

$$\begin{array}{cc} & {\text{VaR}}_{\alpha }\left(\Delta {\overset{ˇ}{S}}_{t}^{\left(i,j\right)}\right)\leq \Phi_{t}^{\left(i,j\right)}, \end{array}$$ (16)

where VaR_*α*_ is defined as:

$$\begin{array}{cc} & {\text{VaR}}_{\alpha }\left(\Delta {\overset{ˇ}{S}}_{t}^{\left(i,j\right)}\right)\text{min}\{\varphi_{t}^{\left(i,j\right)}|P(\Delta {\overset{ˇ}{S}}_{t}^{\left(i,j\right)}\leq \varphi_{t}^{\left(i,j\right)})\geq \alpha \}. \end{array}$$ (17)

VaR_*α*_ refers to the minimum value of the $\Delta {\overset{ˇ}{S}}_{t}^{\left(i,j\right)}$ greater than our model estimation (failing our estimation), happening with probability *α*. The notation $\phi_{t}^{\left(i,j\right)}$ represents a relevant value that can be compared with $\Phi_{t}^{\left(i,j\right)}$.

Nonetheless, to avoid the potential computationally tractability issues brought by Value at Risk [31], we consider another popular performance metric:

$$\begin{array}{c} {\text{CVaR}}_{\alpha }\left(\Delta {\overset{ˇ}{S}}_{t}^{\left(i,j\right)}\right)\leq \Phi_{t}^{\left(i,j\right)}, \end{array}$$ (18)

where CVaR_*α*_ is defined to be the conditional expectations in excess of VaR_*α*_:

$$\begin{array}{c} {\text{CVaR}}_{\alpha }\left(\Delta {\overset{ˇ}{S}}_{t}^{\left(i,j\right)}\right)E[\Delta {\overset{ˇ}{S}}_{t}^{\left(i,j\right)}\;|\;\Delta {\overset{ˇ}{S}}_{t}^{\left(i,j\right)}\geq {\text{VaR}}_{\alpha }\left(\Delta {\overset{ˇ}{S}}_{t}^{\left(i,j\right)}\right)]. \end{array}$$ (19)

On account of our model deciding vaccination via Eq (9), chance constraint (18) enforces more vaccines to be planned. We minimize our estimation $\Phi_{t}^{\left(i,j\right)}$, for its being the best thing we know about $\Delta {\overset{ˇ}{S}}_{t}^{\left(i,j\right)}$. The resulting chance-constraint optimization problem becomes:

$$\begin{array}{cccc} 3\underset{{\left\{v_{t}^{\left(i,j\right)}\right\}}_{t=0}^{T-1}}{\text{min}}\; & \sum_{t=0}^{T-1}\sum_{i=1}^{M}\sum_{j=1}^{K}\Phi_{t}^{\left(i,j\right)} & & \end{array}$$ (20a)

$$\begin{array}{ccc} s.t.\; & X_{t}^{\left(i,j\right)}=p_{t}^{\left(i,j\right)}X_{t}\;X\in \left\{S,E,I,R,C,D,N\right\}, & t=0, \end{array}$$ (20b)

$$\begin{array}{ccc} & \Delta S_{t}^{\left(i,j\right)}=\Delta {\overset{ˇ}{S}}_{t}^{\left(i,j\right)},\Delta {\overset{ˇ}{S}}_{t}^{\left(i,j\right)}∼f_{D}\left(\Delta S_{t}^{\left(i,j\right)}\right),\; & t=0, \end{array}$$ (20c)

$$\begin{array}{ccc} & \text{multi-feature}\;\text{SEIR}\;\text{constraints}\;\text{(}13\text{a})\;-(13\text{j}) & t=1,\cdots ,T-1, \end{array}$$ (20d)

$$\begin{array}{ccc} & V_{t}^{min}\leq \sum_{j=1}^{K}\sum_{i=1}^{M}v_{t}^{\left(i,j\right)}(S_{t}^{\left(i,j\right)}+E_{t}^{\left(i,j\right)}+R_{t}^{\left(i,j\right)})\leq V_{t}^{max}\; & t=0,\cdots ,T-1, \end{array}$$ (20e)

$$\begin{array}{ccc} & v_{t}^{\left(i,j\right)}\in \left[0,1\right] & t=0,\cdots ,T-1, \end{array}$$ (20f)

$$\begin{array}{ccc} & X_{t}^{\left(i,j\right)}\geq 0 & t=0,\cdots ,T-1, \end{array}$$ (20g)

$$\begin{array}{ccc} & {\text{CVaR}}_{\alpha }\left(\Delta {\overset{ˇ}{S}}_{t}^{\left(i,j\right)}\right)\leq \Phi_{t}^{\left(i,j\right)} & t=0. \end{array}$$ (20h)

Due to $\Delta S_{t}^{\left(i,j\right)}$ being stochastic, we generate a realized value $\Delta {\overset{ˇ}{S}}_{t}^{\left(i,j\right)}$ in Eq (20c). For the other future periods, we use $\Delta S_{t}^{\left(i,j\right)}=\Phi_{t}^{\left(i,j\right)}$ as shown in Eq (20d), meaning that we believe future $\Delta S_{t}^{\left(i,j\right)}$ following our estimation. This also creates the difference between actual and estimated value in state S, E, etc. Correspondingly, under such difference, (20h) is introduced to ensure efficiency. We do not consider the difference between $\Delta S_{t}^{\left(i,j\right)}$ and $\Phi_{t}^{\left(i,j\right)}$ for the future (*t* ≥ 1), since they are unrealized. Thus, we still have $\Delta S_{t}^{\left(i,j\right)}=\Phi_{t}^{\left(i,j\right)}$ for *t* = 1, ⋯, *T* − 1. However, in our heuristic solution approach that we discuss later, we can apply the Optimization for small *T* repeatedly, i.e., solve for *T* = 4 sequentially. This will continuously consider the difference between the actual and estimated value of $\Delta S_{t}^{\left(i,j\right)}$ and the population in all states for a future time.

For computation purposes, we formulate the CVaR constraint Eq (20h) explicitly by applying the result in [32]:

$$\begin{array}{cc} \int_{0}^{\varphi_{t}^{\left(i,j\right)}} & f_{D}\left(s\right)\;ds\geq \alpha , \end{array}$$ (21a)

$$\begin{array}{cc} & \varphi_{t}^{\left(i,j\right)}+\frac{1}{1-\alpha }E[z_{t}^{\left(i,j\right)}]\leq \Phi_{t}^{\left(i,j\right)}, \end{array}$$ (21b)

$$\begin{array}{cc} & z_{t}^{\left(i,j\right)}=\text{max}\{0,\;\Delta {\overset{ˇ}{S}}_{t}^{\left(i,j\right)}-\varphi_{t}^{\left(i,j\right)}\}. \end{array}$$ (21c)

We use $\phi_{t}^{\left(i,j\right)}$ to represent ${\text{VaR}}_{\alpha }\left(\Delta {\overset{ˇ}{S}}_{t}^{\left(i,j\right)}\right)$. Eq (21a) follows the definition of VaR_*α*_ in (17). Eqs (21b) and (21c) follows the reformulation of Eq (9.7) in [30, Sect. 2.9]. The expectation in Eq (21b) requires the distribution of $z_{t}^{\left(i,j\right)}$, which depends on $f_{D}\left(\Delta {\overset{ˇ}{S}}_{t}^{\left(i,j\right)}\right)$.

### Intuition-based vaccination and heuristic solutions

Solving the Mixed-Integer-Problem (MIP) reformulation of Optimization (14) and (20) is time-consuming. For many small instances with two different contact rates, two sensitivities, and for *T* = 5, it takes more than 12 hours to get the optimal solution. For efficiency, we propose intuition-based strategies and heuristic solutions to the optimization problem. First, we introduce some intuition-based strategies, prioritizing vaccines based on contact rate and sensitivity. Then, we discuss a heuristic solution, a modified greedy algorithm that sequentially solves the optimization problem in shorter periods.

First, we consider three intuition-based vaccine prioritization strategies for different groups of people based on their contact rate and sensitivity. The first one is noted as C*S, vaccination considering contact rate and sensitivity simultaneously. We decide which (*i*, *j*) division gets vaccinated first, based on the value of *c*^*j*^ ⋅ *s*^*i*^ (contact rate times sensitivity). The larger it is, the higher priority the division has. We choose sensitivity *s* = *γ*, the exposed-infected rate, when prioritizing the vaccine. The second one is noted as S1C2, vaccination considering sensitivity as the priority and contact rate secondly if two people have the same sensitivity. This is the most related to the current protocol, where younger and older people with higher risk get vaccinated first. The third one is noted as C1S2, vaccination considering contact rate as the priority and sensitivity secondly if there is a tie in contact rate. We reverse the order to see if there is an improvement.

Additionally, we use a heuristic solution, a modified greedy algorithm to heuristically solve the optimization. We denote the solution of static Optimization (14) as “Static”, and the solution of chance-constraint Optimization (20) with Eq (21) as “Stochastic”. Each optimization is solved sequentially with a short time slot of *T* = *T*_*g*_ periods. Take *T*_*g*_ = 4 as an example, we solve each optimization for *t* = 0, 1, 2, 3 together to decide the arrangement for the first week. For the second week, we solve each optimization for *t* = 1, 2, 3, 4 together.

## Results

We utilize the multi-feature SEIR model, Optimization (14) and (20) to conduct several numerical experiments evaluating vaccination prioritization strategies. Many of the parameters of our experiments are chosen based on the COVID epidemic/pandemic [3, 5, 19].

This section is organized as follows. In the first subsection *comparison with actual confirmed cases*, to show the usefulness of our model, we compare the confirmed COVID infections from CDC with the estimated infections using multi-feature SEIR and the classic SEIR model. Afterward, in subsection *numerical settings and evaluation metric*, we give a numerical choice for sensitivity, contact rate, population, etc., and the performance metric for vaccination strategy evaluation. In subsection *comparison of intuition-based vaccination*, to select the best intuition-based strategy, we evaluate the performance of different vaccination strategies and benchmark them under different situations. In the last subsection *comparison of heuristic solutions for optimization problems*, we compare the intuition-based strategies with the heuristic solutions of the optimization problems under a severe situation.

### Comparison with actual confirmed cases

To validate our model, we leverage the COVID data for confirmed cases in Allegheny County, Pennsylvania, USA and Hamilton County, Ohio, USA sourced from the CDC [33] and the USAFACTS dataset [34]. We then compare this data with the estimated infection cases derived from both the multi-feature SEIR and classic SEIR models.

**Fig 4:**
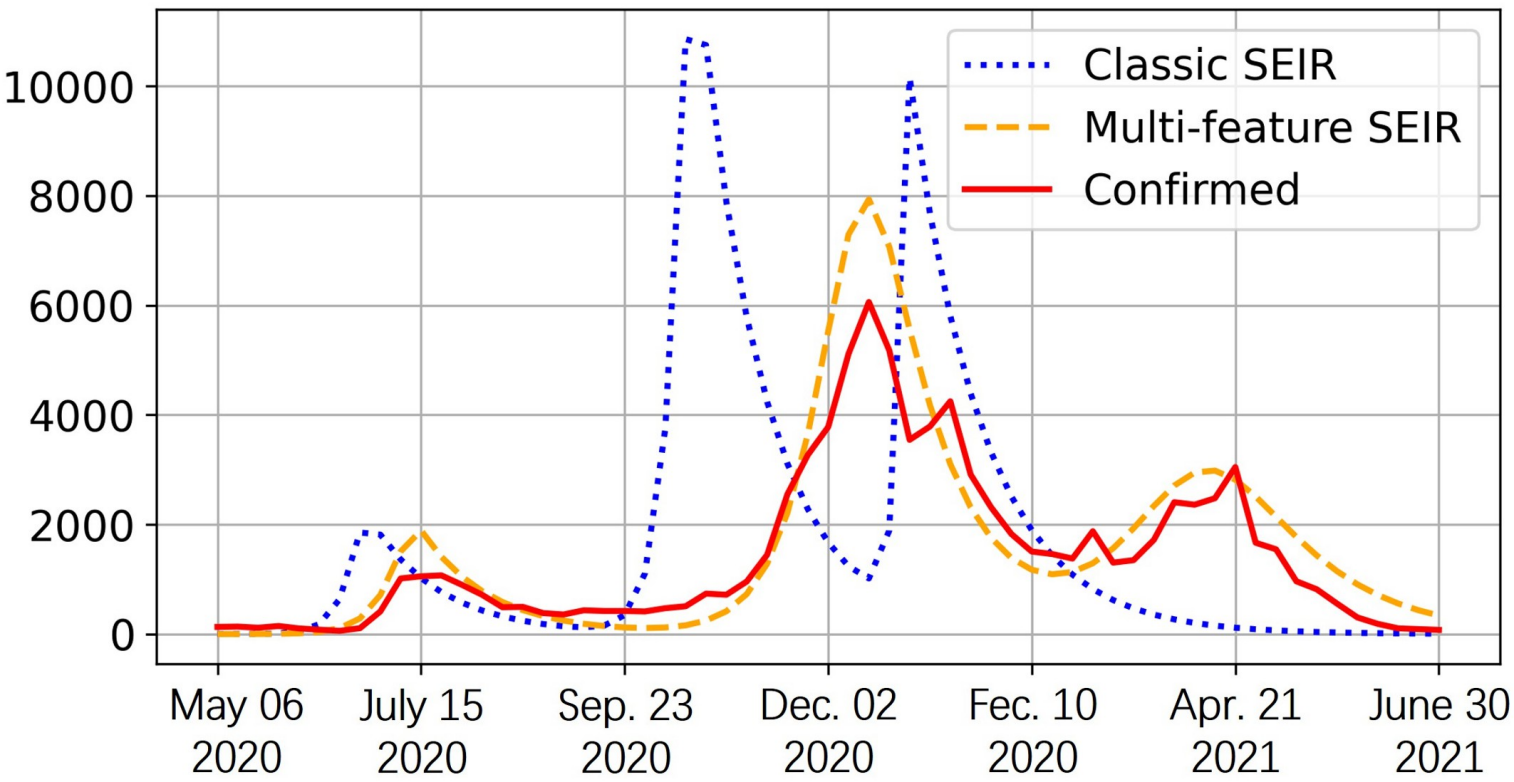
Weekly COVID confirmed and estimated cases of new infection in Allegheny County. The vertical axis is the infected population. We compare the estimation of the infected population using historical data among the classic SEIR model, our proposed multi-feature SEIR, and actual confirmed infection cases. The observation period concludes on June 30th, 2021.

Our choice for the starting time is approximately when the confirmed cases have reached a substantial level. The endpoint is selected to be approximately the end of the last wave preceding the widespread vaccination distribution. As a result, the time period considered for Allegheny County spans from early May 2020 to June 2021, while for Hamilton County, it spans from late March 2020 to the beginning of July 2021. In Fig 4, the confirmed case shows three waves of the pandemic in Allegheny County. To align with reality, we consider three stages for the spread different sub-population is assigned to each stage based on the total confirmed cases and related regulations. The first stage begins in late April 2020, when the spread of COVID was about to start again (new daily confirmed cases started to rise after the time of decline). The second stage begins in the middle of August when the new confirmed cases become stable. The last stage begins in late October 2020, when the new confirmed cases started to rise again. The beginning time of these stages is referred to historical data but can also be decided based on the medical prediction of the next coming wave. For each of them, we fit the parameters for the best estimation.

**Fig 5:**
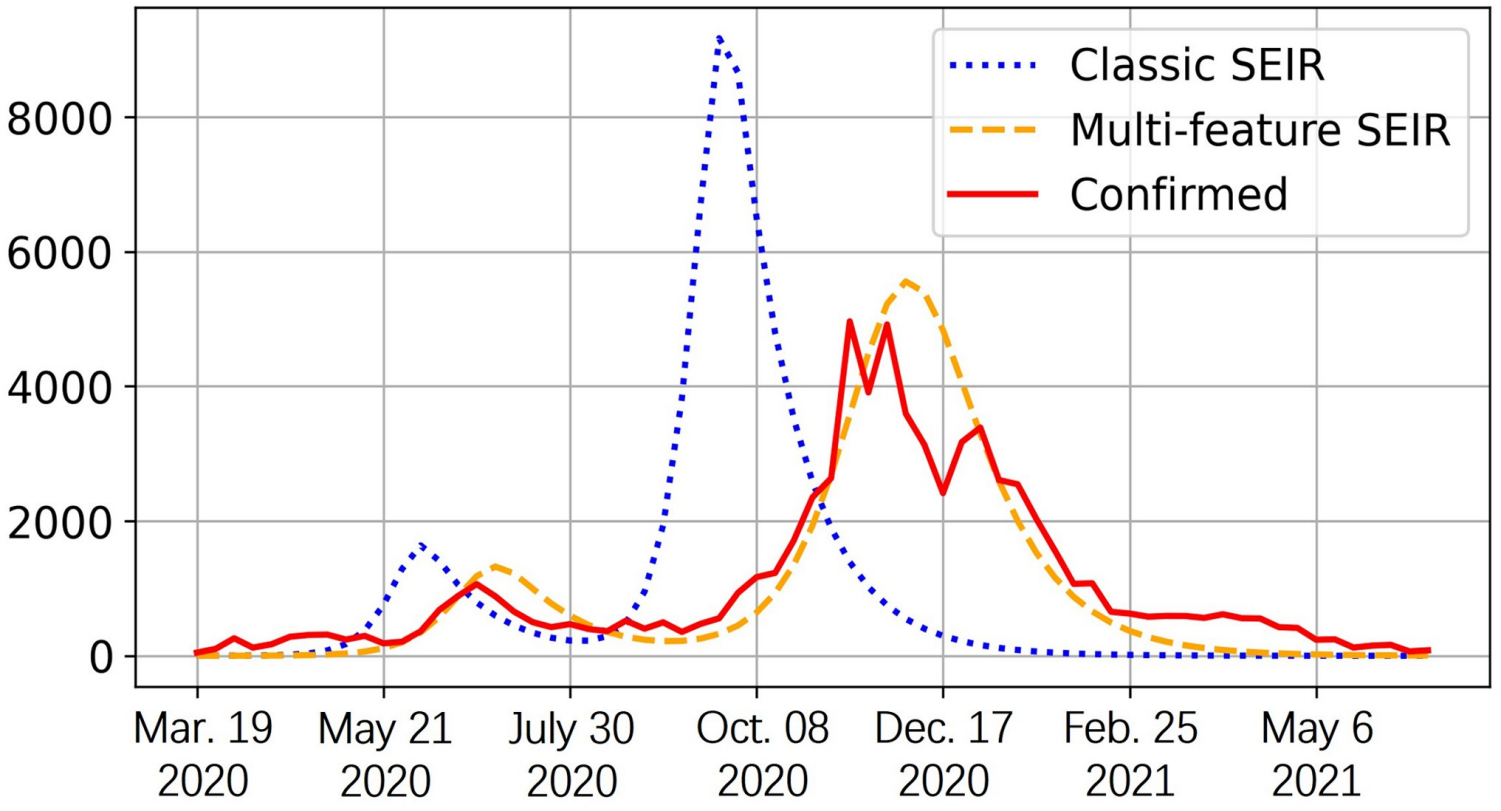
Weekly COVID confirmed and estimated cases of new infection in Hamilton County. The vertical axis is the infected population. We compare the estimates of the infected population using the classic SEIR model, our proposed multi-feature SEIR model, and the actual confirmed infection cases. The observation period concludes on July 5th, 2021.

In Fig 5, the confirmed cases exhibit two distinct waves of the pandemic in Hamilton County. We divide the pandemic into two stages, each with different sub-populations assigned to it. The first stage commenced in late May 2020, marked by the increasing severity of COVID spread. The second stage began in July when a new outbreak started.

For both datasets, we measure the accuracy of both SEIR models by the following estimation error *ϵ*. Here, *I*_*k*_ represents the number of week *k* infections.

$$\begin{array}{c} \epsilon =\sum_{k}|\text{Confirmed}\;I_{k}-\text{Estimated}\;I_{k}|. \end{array}$$

As we observed, the prediction of multi-feature SEIR aligns with the actual data much more than the classic model. Classic SEIR does not accurately identify the pandemic pattern and predicts the peak much earlier, and our multi-feature SEIR predicts the patterns and peaks much more accurately. We also see that the estimated infection of multi-feature SEIR is slightly higher than the confirmed cases most of the time. This is because the confirmed cases only include the reported ones and underestimated the actual number. The number of total infections can be higher than the number of total confirmed infections [35]. In addition, we quantified performance using the estimation error, *ϵ*, by considering the estimated weekly infection population and confirmed weekly infections in Table 2. The findings indicate the superiority of our proposed multi-feature SEIR model.

**Table 2:** Comparison of estimation error measured by *ϵ* by considering weekly infected populations between classic and multi-feature SEIR.

County	*ϵ* for classic SEIR	*ϵ* for multi-feature SEIR
Allegheny	97589.9129	30207.3016
Hamilton	89138.8527	25059.0885

### Numerical settings and evaluation metric

Next, we introduce the settings of population parameters, as well as performance metrics in vaccination strategy evaluation.

#### Sensitivity (*s*)

Sensitivity represents vulnerability, indicating how likely a person will transit to another disease state. We utilize a common measurement of sensitivity, the rate of transition between states, and these rates are selected based on the actual duration a person spends in a specific state [1–3]. Different sensitivities are applied to different states a person may occupy. For our simulation, we divide all sensitivities into two groups: one with higher sensitivity (greater vulnerability) and one with lower sensitivity. Table 3 provides the range of choices based on the duration data presented in [3].

**Table 3:** Sensitivity parameters and values in SEIR model.

Parameters	Symbols	Value	Description
Infection probability	λ	0.2	Probability of infection
Exposed to infected rate	*γ*	1/14∼1/5	5 to 14 days incubation period
Recovery rate (exposed)	*σ*	1/14	14 days quarantine period
Recovery rate (infected)	*θ*	1/20∼1/10	10 to 20 days to recovery for *I*
Death rate	*δ*	2.3% − 2.6%	Case fatality rate

Based on the table, we choose the value of high sensitivity to be *s*^*h*^ ∈ {0.2, 0.14}, and low sensitivity to be *s*^*l*^ ∈ {0.1, 0.07}.

#### Contact rate (*c*)

For our simulation, we set two contact rate groups based on the simulations in [26]. High contact rate is from {25, 20, 15, 10} and low contact rate from {15, 10, 5}. The contact rate of the first group is always higher than the second group.

*Proportion* (*p*^*s*^, *p*^*c*^): We set many situations for the initial proportion of the two sensitivity groups: *p*^*s*^ = (*p*^*s*, 1^, *p*^*s*, 2^), and the two contact rate groups: *p*^*c*^ = (*p*^*c*, 1^, *p*^*c*, 2^). Both vary from (0.95, 0.05) to (0.05, 0.95) with a 0.1 increment. (0.95, 0.05) to (0.55, 0.45) and (0.45, 0.55) to (0.05, 0.95) are classified as “High” and “Low” situation, respectively.

#### Initial exposed population

To see how the initial exposed population affects the result, we set the initial exposed population to take up *α* proportion of the susceptible population, *α* ∈ {0.1%, 0.2%, 0.5%, 1%}. The lower the value of *α* is, the less severe the outbreak will be. States other than susceptible and exposed are zero at the beginning.

#### Vaccine amount

The maximum amount of vaccines in one period is decided by *V*_total_/*T*_*c*_, where *V*_total_ is the total amount of vaccines available during the whole time we consider, and *T*_*c*_ represents the total time planned to vaccinate the whole population (vaccine is evenly distributed for each period). We estimate *V*_total_ by the initial total population and consider multiple vaccine doses. We set *T*_*c*_ = 100 weeks, representing nearly two years.

#### Parameter selection

For experiments, we choose the sensitivity based on the actual duration a person spends in a specific state [3]. The contact rate can be chosen based on the particular social network. We chose the contact rate from the social network simulation results in [27]. The proportion values, initial exposed population, and vaccine amount can be varied and tailored to fit specific scenarios and populations. For example, the initial exposed population can be available after observing a disease outbreak.

#### Performance metrics

The effectiveness of the vaccination strategy is measured in terms of infection population and cumulative death. To measure cumulative death, we use *death proportion*. It is the ratio of cumulative death to the total initial population. Besides, we compute the *average loss ratio* for each strategy over all non-winning cases. We define the *loss ratio* of the highest infection population as follows:

$$\begin{array}{c} \text{Loss}\;\text{ratio}\;\text{of}\;\text{highest}\;I_{t}=\frac{\max_{0\leq t\leq T}\left\{I_{t}\right\}\;\text{of}\;\text{given}\;\text{strategy}-\max_{0\leq t\leq T}\left\{I_{t}\right\}\;\text{of}\;\text{the}\;\text{best}\;\text{strategy}}{\max_{0\leq t\leq T}\left\{I_{t}\right\}\;\text{of}\;\text{given}\;\text{strategy}}. \end{array}$$

We do the same to define the loss ratio of cumulative death.

### Comparison of intuition-based vaccination

In this subsection, we conduct simulations using populations with constant parameters over time. Even though the reality is usually time-varying, we use static simulation to provide suggestions for a short period. We compare the strategies under 8000 different situations of population characteristics. Lastly, our study provides statistical support for the efficacy of S1C2 vaccination strategy.

#### Static simulation for effectiveness comparison

As we discussed before, we consider the performance in two aspects, highest infection and cumulative death. We study the effectiveness of different vaccination strategies by the winning rate of the highest infection proportion and death proportion for each aspect. We also summarize the average loss ratio to analyze the gap compared to the best strategy. Through the simulations for 8000 situations of population characteristics, we observe that the effectiveness of strategy heavily depends on the severity of the epidemic/pandemic. Thus, we only compare the strategies under similar severeness. The result shows that S1C2 strategy performs the best in most cases, but C*S and C1S2 can be better under severe situations, where high contact rate and high sensitivity people take at least 50% of the total population.

Below we summarize the performance of each strategy based on the different severeness of the situation. The severeness is modeled by the initial proportion of the high contact rate population and high sensitivity population. Four situations are considered: 1) *High-High* refers to severe situations, where the first *High* refers to people with high sensitivity taking up the majority (>50% of the total population), and the second *High* refers to people with high contact rate taking up the majority of the total population; 2) *Low-High* refers to the situation where low sensitivity people take up the majority in terms of sensitivity, and high contact rate people is more than 50%; 3) *High-Low* refers to the situation where high sensitivity people is more than 50%, and low contact rate people is more than 50%; 4) *Low-Low* refers to the situation where both low sensitivity people and low contact rate people take up the majority of the total population. Each situation contains 2000 simulations (4 initial exposed proportions, 4 sensitivities, 5 contact rates, 5 sensitivity proportions, and 5 contact rate proportions).

In the following, we compare the *winning rate* and *average loss ratio* for each situation. The winning rate counts the percentage of winning in terms of the two metrics (highest infection proportion and death proportion) among 2000 cases. The average loss ratio is defined at the beginning of result section, indicating the difference to the best strategy. Tables 4 and 5 exhibit the winning rate of each strategy in terms of highest infection proportion and death proportion, respectively. Tables 6 and 7 shows the average loss in terms of highest infection and cumulative death population, respectively. Strategy C_only and Random are omitted for they do not win under any situation.

**Table 4:** Winning rate of highest infection proportion of each strategy under different situations.

Situation	C*S	S1C2	C1S2
High-High	100.00%	100.00%	100.00%
Low-High	87.25%	99.95%	73.50%
High-Low	83.90%	97.20%	71.35%
Low-Low	62.50%	97.95%	26.45%

**Table 5:** Average loss ratio in highest infection of each strategy under different situations.

Situation	C*S	S1C2	C1S2
High-High	0.00%	0.00%	0.00%
Low-High	0.58%	0.01%	0.62%
High-Low	0.90%	0.98%	0.81%
Low-Low	2.20%	1.56%	2.18%

**Table 6:** Winning rate of the death proportion of each strategy under different situations.

Situation	C*S	S1C2	C1S2
High-High	44.00%	8.10%	91.90%
Low-High	44.00%	68.90%	26.35%
High-Low	41.35%	76.05%	17.95%
Low-Low	48.15%	96.45%	1.65%

**Table 7:** Average loss ratio in cumulative death of each strategy under different situations.

Situation	C*S	S1C2	C1S2
High-High	0.08%	0.07%	0.04%
Low-High	0.45%	0.07%	0.82%
High-Low	0.61%	0.22%	0.89%
Low-Low	3.24%	1.22%	3.66%

In Table 4, each percentage represents the winning chance of having the lowest value in the highest infection proportion among 2000 simulated cases. S1C2 performs the best in all situations, winning more than 97% of cases in terms of the highest infection proportion. The hundred percent under High-High situation in the first row of Table 4 means that all three strategies perform the same. Under other situations, C*S and C1S2 are worsening (winning rate decreases to 62.50% and 26.45%, respectively). The summation of three strategies is over 100%, meaning that some situations have multiple best strategies.

For cases where a strategy does not win in the highest infection proportion, we calculate the average loss ratio to investigate the difference with the best strategy in Table 6. The average is taken over all non-winning cases among 2000 simulated cases. Most percentages are less than 1%, showing a small loss to the best strategy. This also shows that a low winning rate does not necessarily mean poor performance. Hence, S1C2 is still the most reliable strategy, with a high winning chance and small average loss when it is not the best strategy.

We further evaluate the performance in terms of cumulative death in Tables 5 and 7, which is also proportional to the cumulative number of infections.

In Table 5, each percentage represents the winning probability of having the lowest value in death proportion among 2000 simulated cases. Under severe situations (High-High), C1S2 performs the best, and C*S is also better than S1C2. In other situations, the S1C2 strategy is the best. When the situation is getting less severe (moving vertically along the situation column), S1C2 has an increasing winning chance. This indicates the superiority of S1C2 in unsevere situations, where low sensitivity or low contact rate people take the majority.

For cases where a strategy does not win in terms of death proportion, we calculate the average loss ratio to investigate its difference to the best strategy in Table 7. The average is taken over all non-winning cases among 2000 simulated cases. Similarly, most percentages are less than 1%, showing a small loss to the best strategy. Note that under the High-High situation, the average loss for S1C2 is only 0.07%. In conclusion, C1S2 is preferred under severe (High-High) situations. In circumstances where we do not know the severity of the situation, S1C2 is suggested because its difference to the best strategy is marginally small under unfavorable situations.

#### Static simulation for severe situation

In Table 6, each percentage represents the winning probability of having the lowest value in death proportion among 2000 simulated cases. Under severe situations (High-High), C1S2 performs the best, and C*S is also better than S1C2. In other situations, the S1C2 strategy is the best. When the situation is getting less severe (moving vertically along the situation column), S1C2 has an increasing winning chance. This indicates the superiority of S1C2 in unsevere situations, where people with low sensitivity or low contact rates take the majority.

For cases where a strategy does not win in terms of death proportion, we calculate the average loss ratio to investigate its difference to the best strategy in Table 7. The average is taken over all non-winning cases among 2000 simulated cases. Similarly, most percentages are less than 1%, showing a small loss to the best strategy. Note that under the High-High situation, the average loss for S1C2 is only 0.07%. In conclusion, C1S2 is preferred under severe (High-High) situations. In circumstances where we do not know the severity of the situation, S1C2 is suggested because its difference from the best strategy is marginally small under unfavorable situations.

In Tables 8 and 9, we have four situations, and each rate is computed from 500 cases. All four situations have a similar pattern to the High-High situation in Tables 6 and 7. This indicates that the initial proportion of population does not affect the performance of a strategy. In Tables 10 and 11, for each sensitivity situations, we consider 500 cases to compute each percentage. C1S2 wins the majority of the time. S1C2 has its highest winning chance with sensitivity *γ* = (0.14, 0.1), and it is largely different from other cases. The total winning rate of S1C2 and C1S2 is 100%. In Tables 12 and 13, for each contact rate cases, we have 400 cases to compute each percentage. C1S2 has the highest winning rate, and the total winning rate of S1C2 and C1S2 is 100% as well. Through these comparisons, we witness that the initial proportion of population does not have much impact on the effectiveness of intuition-based strategies. In contrast, sensitivity and contact rate have more influence. Meanwhile, C1S2 works the best under severe situations, regardless of different population features.

**Table 8:** Winning rate in death proportions under High-High situations.

Initial %*E*	C*S	S1C2	C1S2
0.1%	43.80%	9.00%	91.00%
0.2%	44.00%	8.40%	91.60%
0.5%	43.80%	7.80%	92.20%
1%	44.40%	7.20%	92.80%

**Table 9:** Average loss ratio in cumulative death under High-High situations.

Initial %*E*	C*S	S1C2	C1S2
0.1%	0.08%	0.07%	0.05%
0.2%	0.08%	0.07%	0.04%
0.5%	0.08%	0.07%	0.04%
1%	0.08%	0.07%	0.03%

**Table 10:** Winning rate in death proportion of each strategy under High-High situations.

Sensitivity	C*S	S1C2	C1S2
(0.2, 0.1)	40.20%	0.20%	99.80%
(0.14, 0.1)	73.60%	26.40%	73.60%
(0.2, 0.07)	20.20%	0.20%	99.80%
(0.14, 0.07)	42.00%	5.60%	94.40%

**Table 11:** Average loss ratio in cumulative death of each strategy under High-High situations.

Sensitivity	C*S	S1C2	C1S2
(0.2, 0.1)	0.08%	0.08%	0.01%
(0.14, 0.1)	0.06%	0.01%	0.06%
(0.2, 0.07)	0.13%	0.13%	0.01%
(0.14, 0.07)	0.05%	0.05%	0.09%

**Table 12:** Winning rate in death proportion of each strategy under High-High situations.

Contact	C*S	S1C2	C1S2
(25, 15)	19.75%	7.25%	92.75%
(25, 10)	68.00%	7.00%	93.00%
(20, 10)	20.00%	7.50%	92.50%
(20, 5)	92.25%	7.75%	92.25%
(10, 5)	20.00%	11.00%	89.00%

**Table 13:** Average loss ratio in cumulative death of each strategy under High-High situations.

Contact	C*S	S1C2	C1S2
(25, 15)	0.08%	0.07%	0.06%
(25, 10)	0.11%	0.07%	0.06%
(20, 10)	0.08%	0.07%	0.06%
(20, 5)	0.06%	0.07%	0.06%
(10, 5)	0.08%	0.07%	0.07%

### Comparison of heuristic solutions for optimization problems

In this subsection, we evaluate the performance of heuristic solutions and intuition-based strategies, considering the uncertainty of disease transmission (influence on $\Delta S_{t}^{\left(i,j\right)}$). First, we give the distribution of $\Delta S_{t}^{\left(i,j\right)}$. Next, simulations are conducted, and the performance of all solutions is compared. Furthermore, the results highlight the robustness of our chance-constraint formulation, affirming its effectiveness in scenarios with uncertainty.

In the following numerical study, we define the distribution of $\Delta S_{t}^{\left(i,j\right)}$ and $f_{D}\left(\Delta S_{t}^{\left(i,j\right)}\right)$ as discrete distribution in Table 14, with given support centered around our estimation $\Phi_{t}^{\left(i,j\right)}$:

**Table 14:** Example of discrete distributions for $\Delta S_{t}^{\left(i,j\right)}$.

Value		$0.9\Phi_{t}^{\left(i,j\right)}$	$0.95\Phi_{t}^{\left(i,j\right)}$	$\Phi_{t}^{\left(i,j\right)}$	$1.05\Phi_{t}^{\left(i,j\right)}$	$1.1\Phi_{t}^{\left(i,j\right)}$
Proability	Situation 1	0	0	1	0	0
	Situation 2	0.01	0.1	0.78	0.1	0.01
	Situation 3	0.05	0.2	0.5	0.2	0.05

The discrete distribution make the integral and expectation in Eq (21) easy to compute. Note that for the first period of Optimization (14) and (20), we randomly generate a value for $\Delta {\overset{ˇ}{S}}_{t}^{\left(i,j\right)}$ for each division. Since we have 4 divisions in the experiment, they are generated independently. Thus, our estimation may underestimate the real situation for some (*i*, *j*) divisions, but is effective for other divisions.

We use heuristic solutions for Optimization (14) and (20). Solution of static Optimization (14) is noted as “Static”. Solution of Optimization (20) with Eq (21) is called “Stochastic”. For details of the heuristic solution, please refer to the section on *intuition-based vaccination and heuristic solutions*.

Due to $\Delta S_{t}^{\left(i,j\right)}$ being stochastic, we generate its value $\Delta {\overset{ˇ}{S}}_{t}^{\left(i,j\right)}$ according to the distributions in Table 14 for the first current period. For the other future periods, we use $\Delta S_{t}^{\left(i,j\right)}=\Phi_{t}^{\left(i,j\right)}$, meaning that we believe future $\Delta S_{t}^{\left(i,j\right)}$ still following our estimation. Note that the *Static* strategy still uses $\Delta S_{t}^{\left(i,j\right)}=\Phi_{t}^{\left(i,j\right)}$ for the first period, regardless of the realized value being different from $\Phi_{t}^{\left(i,j\right)}$. While the *Stochastic* solution is aware of such difference, with an extra Constraint (20h).

#### Comparison between intuition-based and heuristic solutions

Lastly, we compare intuition-based strategies and heuristic solutions under one severe situation. To measure the performance of each strategy, we consider the highest exposed, infected population over time and the cumulative death as the performance metric. We also take the average from 20 simulations for each situation. We set *α* = 0.95 in Constraint (20h).

We find that both heuristic solutions are better than intuition-based ones. Moreover, with the uncertainty in $\Delta S_{t}^{\left(i,j\right)}$, the Stochastic solution from Optimization (20) is more efficient than the Static solution from Optimization (14). However, in some scenarios, intuition-based strategies can be as effective as the modified greedy algorithm and is less time-consuming.

**Fig 6:**
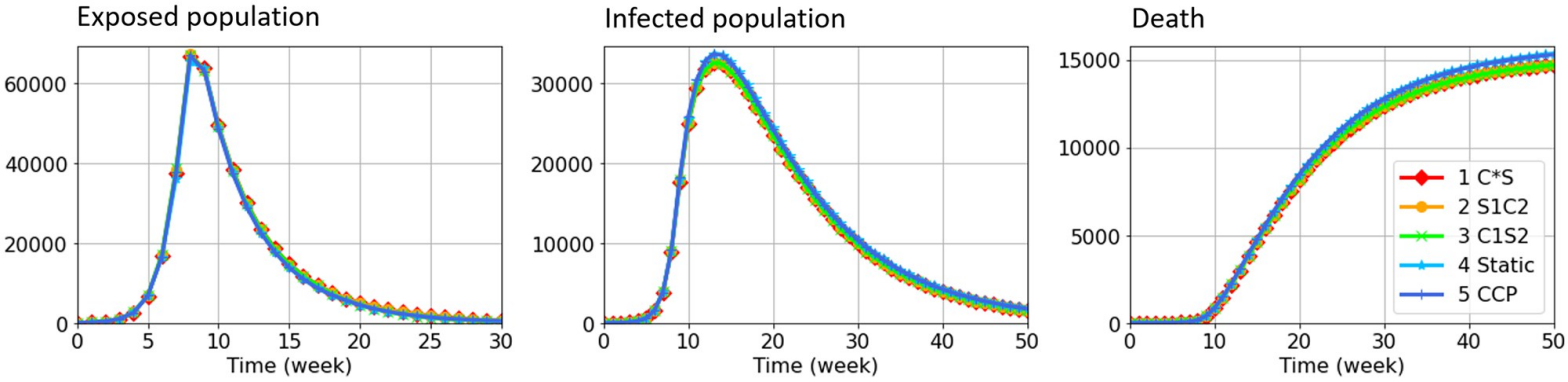
Population change of exposed, infected, and dead state in situation 3 using intuition-based strategies and approximated optimal strategies. The population changes under given parameters and different vaccination strategies. All strategies perform similarly. Situation 1 and 2 present observably indifferent results, so only Situation 3 is presented.

Fig 6 compares the population change under situation 3 in Table 14. Population changes in other situations are observably indifferent. The horizontal axis is time (week), and the vertical axis is the population in a given state. We set the initial population as *S*_0_ = 100000, *E*_0_ = 50. The population of other states is zero. The two-group sensitivities for different states are λ = (0.1, 0.1), *γ* = (0.2, 0.1), *σ*^*E*^ = (1/14, 1/14), *σ*^*I*^ = (1/20, 1/10), *δ* = (0.025, 0.025), with initial proportion $p_{0}^{s}=\left(0.5,0.5\right)$. The two-group contact rates are *c* = (25, 15), with initial proportion $p_{0}^{c}=\left(0.5,0.5\right)$. We consider a period of 50 weeks. Vaccination strategies are intuition-based vaccination strategies (C*S, S1C2, C1S2) and heuristic solutions (*Static* and *Stochastic*, noted as CCP for short). For *Static* and *Stochastic* (CCP) solutions, we approximated the optimal solution by modified greedy solution with *T*_*g*_ = 4, which sequentially solves the optimization for *T* = 4 periods (one month). The solutions are provided by the Gurobi solver. Some of the performances are listed in Tables 15 to 17.

**Table 15:** Performance of strategies in situation 1.

Performance	Intuition-based Strategy			Heuristic Solution	
	C*S	S1C2	C1S2	Static (14)	Stochastic (20)
Highest *E*	67026.91	67026.91	67026.91	66389.16	**66389.16**
Highest *I*	32596.55	32596.55	32596.55	33747.64	**33747.64**
Cumulative *D*	14710.88	14710.88	14685.72	15324.36	**15324.36**
Time (seconds)	0.0684	0.0782	0.0641	5.1378	5.4507

**Table 16:** Performance of strategies in situation 2.

Performance	Intuition-based Strategy			Heuristic Solution	
	C*S	S1C2	C1S2	Static (14)	Stochastic (20)
Highest *E*	67004.31	66985.55	**66957.03**	66614.27	**66102.33**
Highest *I*	32596.00	32595.53	**32594.81**	34002.18	**33830.14**
Cumulative *D*	14710.73	14710.59	**14685.24**	15528.57	**15433.14**
Time (seconds)	0.0617	0.0744	0.0655	5.5280	5.9965

**Table 17:** Performance of strategies in situation 3.

Performance	Intuition-based Strategy			Heuristic Solution	
	C*S	S1C2	C1S2	Static (14)	Stochastic (20)
Highest *E*	**66859.17**	66906.04	66924.53	66893.48	**66482.49**
Highest *I*	**32592.41**	32593.56	32594.02	33961.19	**33312.36**
Cumulative *D*	14709.71	14710.02	**14685.01**	15482.50	**15122.34**
Time (seconds)	0.0633	0.0801	0.0630	5.9078	6.2561

All strategies perform at a similar level in terms of the exposed population, infection, and cumulative death. We only show the result of situation 3, since performance in other situations is quite the same. To find the best strategy against the uncertainty in $\Delta S_{t}^{\left(i,j\right)}$, we further summarize the average performance metric in Tables 15 to 17 from 20 simulations for each strategy.

Situation 1 is static (no stochasticity in $\Delta S_{t}^{\left(i,j\right)}$), so *Static* and *Stochastic* have the same performance, superior to all intuition-based strategies. In Situation 2 and 3, due to the uncertainty in $\Delta S_{t}^{\left(i,j\right)}$, *Static* solution from Optimization (14) always performs worse than the *Stochastic* solution from Optimization (20). Nonetheless, two heuristic solutions have the lowest *highest exposed population* in all situations compared to intuition-based ones.

Since the objective function is minimizing all $\Phi_{t}^{\left(i,j\right)}$ (our estimation of the increase in exposed population), intuition-based strategies have a better performance in terms of infected and death populations. Besides, their performance in the exposed population is not far from heuristic solutions. More importantly, intuition-based strategies are much faster than solving an optimization problem.

## Discussion

In this paper, we introduced the new multi-feature SEIR model. Based on the numerical studies in subsection *comparison with actual confirmed cases*, the multi-feature SEIR model excels in accurately predicting the trajectory of COVID outbreaks compared to the classical SEIR model in Figs 4 and 5. The estimation error exhibited in Table 2 further confirms the effectiveness of the multi-feature SEIR model.

Based on the multi-feature SEIR model, in subsection *intituition-based vaccination and heuristic solutions*, we provide strategies and heuristics for vaccination prioritization. Then, subsection *comparison of intuition-based vaccination* benchmarks the performance of vaccine prioritization strategies and provides statistical evidence to support the rationale behind the vaccination strategy (S1C2). While S1C2 may not perform optimally in the context of a severe situation (High-High), the statistics in Tables 8 to 13 do not reveal a significant deviation from a superior strategy.

In subsection *comparison of heuristic solutions for optimization problems*, we confirm that the *Stochastic* solutions derived from chance-constraint optimization outperform the *Static* solution for the original optimization model. Lastly, the statistics in Tables 15–17 demonstrate that our designed vaccination prioritization strategies, which are more computationally efficient as they do not require solving optimization problems, perform almost as well as the *heuristic solutions* obtained by solving Optimization (20) models.

## Conclusion

In this study, we propose a novel multi-feature SEIR model that extends the classic SEIR model by incorporating population heterogeneity in both sensitivity and contact rate. Our model offers improved predictive accuracy compared to the classic SEIR model when applied to CDC data on confirmed infection cases. Our multi-feature SEIR model also enables us to develop and evaluate effective vaccination prioritization strategies under different population characteristics. We find that while specific strategies may be optimal in certain situations, the current protocol vaccination strategy performs well in most cases and reasonably well in unfavorable ones. Moreover, we developed a chance constraint version of our model that takes into account the possibility of estimation failure and maintains the efficiency of the vaccination prioritization strategy. While our focus in this paper is on vaccination as an intervention, our framework can be extended to the combination of multiple intervention approaches, including testing, vaccination, social distancing, and others. In future work, we plan to incorporate additional population features into our model and evaluate more intervention strategies.

## Supporting information

S1 File This file contains the performance metrics (highest infection and cumulative death) of different vaccination strategies for four situations (High-High, Low-High, High-Low, Low-Low). Statistics in Tables 4 to 7 are computed based on this file. Statistics in Tables 8 to 13 are computed based on the first spreadsheet in this file.
(XLSX)
